# Epidemiology and Genetic Variabilities of Human Adenovirus Type 55 Reveal Relative Genome Stability Across Time and Geographic Space in China

**DOI:** 10.3389/fmicb.2020.606195

**Published:** 2020-12-02

**Authors:** Shi-ying Chen, Wenkuan Liu, Yun Xu, Shuyan Qiu, Yong Chen, Xingui Tian, Rong Zhou

**Affiliations:** State Key Laboratory of Respiratory Disease, National Clinical Research Center for Respiratory Disease, Guangzhou Institute of Respiratory Health, The First Affiliated Hospital of Guangzhou Medical University, Guangzhou Medical University, Guangzhou, China

**Keywords:** human adenovirus type 55, acute respiratory disease (ARD), mutation, comparative genomics, virulence, China

## Abstract

After the first outbreak in China in 2006, human adenovirus type 55 (HAdV-B55) has become a common pathogen causing life threatening pneumonia in northern China. However, HAdV-B55 infection has been rarely reported in southern China. Here, we collected throat swabs from 3,192 hospitalized children with acute respiratory disease (ARD) from May 2017 to April 2019 in Guangzhou, southern China, tested them for HAdV-B55 infection. Only one of 1,399 patients from May 2017 to April 2018 was HAdV-B55 positive; HAdV-B55 infections significantly increased with 10 of 1,792 patients testing positive since May 2018. HAdV-B55-267, isolated from a case of death, was sequenced for whole genomic analysis. Three other strains, HAdV-B55-Y16, -TY12, and -TY26, isolated earlier in patients from Shanxi, northern China, were also sequenced and analyzed. The four HAdV-B55 strains formed similar plaques, grew to similar titers, and resulted in similar typical cell pathogenic effects. HAdV-B55-267 formed a subclade with the prototype strain QS-DLL; strains HAdV-B55-Y16, -TY12, and -TY26 were closely related to strain QZ01. HAdV-B55 could be divided into two subtypes (HAdV-B55-a and -b) according to the presence or absence of the insertion of “CCATATCCGTGTT”; all strains isolated from China except for strain BJ01 belong to subtype b. HAdV-B55-267 had only one non-synonymous substitution comparing with strain QS-DLL, and all HAdV-B55 strains had highly conserved capsid proteins and few non-synonymous substitutions. This study suggests that HAdV-B55 is an important pathogen associated with ARD in Guangzhou since 2018, exhibiting the relative genome stability across time and geographic space in China.

## Introduction

Human adenovirus type 55 (HAdV-B55) is a newly identified adenovirus type that causes severe pneumonia. HAdV-B55 was previously identified as HAdV-B11a, which was first isolated from a military trainee during an outbreak in Spain in 1969 (Hierholzer et al., 1974). It re-emerged in the United States in 1997, of which only the capsid proteins gene sequences have been reported (News From the Centers for Disease Control and Prevention, 1998), and then in Egypt in 2001 (GenBank NO. JX423385). Since then, this virus has circulated in South America (Kajon et al., 2013), Europe (Lafolie et al., 2016), and Asia (Chmielewicz et al., 2005; Yang et al., 2009; Zhu et al., 2009; Kajon et al., 2010a; Li et al., 2014; Lu et al., 2014; Sun et al., 2014; Salama et al., 2016; Gu et al., 2017; Wang et al., 2017; Yi et al., 2017; Cheng et al., 2018; Xu et al., 2018; Huh et al., 2019; Jing et al., 2019). In 2006, the first isolated HAdV-B55 strain QS-DLL, was reported as the cause of acute respiratory disease (ARD) in China (Yang et al., 2009; Zhu et al., 2009). Since then, HAdV-B55 has widely spread among the military and public in many provinces across China and has become one of the important pathogens causing pneumonia in the country (Zhang et al., 2012, 2016; Cao et al., 2014; Li et al., 2014; Lu et al., 2014; Sun et al., 2014; Wang et al., 2017; Yi et al., 2017; Jing et al., 2019). Due to the lack of population immunization against HAdV-B55, this virus is likely to spread widely and lead to severe disease epidemics (Zheng et al., 2017). Moreover, compared with other types of adenoviral infections, the symptoms of pneumonia caused by HAdV-B55 infection are usually more serious (Cao et al., 2014).

HAdV-B55 has become an important pathogen associated with ARD in children in many regions of China in recent years, but especially in northern China. However, HAdV-B55 infections were rarely reported in southern China including the city of Guangzhou, the provincial capital of Guangdong; thus, it was not known whether HAdV-B55 was an important pathogen in Guangzhou or not. Therefore, it was necessary to investigate the molecular epidemiology of HAdV-B55 strain in Guangzhou, especially since 14 years had passed since the first strain was reported in China. It is very like that during this time, the genome of HAdV-B55 evolved with mutations that may impact its virulence.

HAdV-B55 evolved via a homologous recombination between HAdV-B11 and HAdV-B14, with the HAdV-B11 hexon gene inserted into the HAdV-B14 genome (Walsh et al., 2010; Zhang et al., 2012; Seto et al., 2013). Since HAdV-B55 can be partially neutralized by HAdV-B11 antiserum, it had previously been named HAdV-B11a (Hierholzer and Pumarola, 1976; Walsh et al., 2010; Seto et al., 2013; Liu et al., 2014). Today, the availability of high-resolution genomic data has provided insights into the molecular evolution of human adenoviruses. Comparative analysis of such adenovirus genomic data is a highly quantitative, cost-effective, expedient, and reliable approach for adenovirus classification, making this approach highly preferred for such purposes (Madisch et al., 2005; Seto et al., 2011; Singh et al., 2012). Because this approach uses primary sequence data, the evolutionary relationships among viruses can be accurately identified since this approach uses the primary sequence data.

In this study, we collected throat swabs from 3,191 hospitalized children with ARD from May 2017 to April 2019 in Guangzhou, southern China, to test for HAdV-B55 infection using real-time polymerase chain reaction (PCR). HAdV-B55-267, was isolated from a child who presented with severe ARD and later died in June 2018 and sequenced for whole genomic analysis along with three other strains, HAdV-B55-Y16, -TY12, and -TY26, isolated earlier in patients from northern China. Bioinformatics and comparative genomic analyses were conducted using these strains and all of the other HAdV-B55 strains listed in GenBank. In addition, the *in vitro* growth of these strains was also analyzed and compared. Our data provide a high-resolution view of the highly similar yet intriguingly divergent genomes of HAdV-B55 strains, thereby elucidating their evolution, and contributing to the prevention and management of HAdV-B55 infections in the future.

## Materials and Methods

### Sample Collection and Real-Time PCR

Throat swab samples from hospitalized pediatric patients (≤17 years old) with ARD were collected at the First Affiliated Hospital of Guangzhou Medical University or Guangzhou Women and Children’s Medical Center from May 2017 to April 2019. The samples were collected and refrigerated at 2–8°C in viral transport medium according to our established clinical protocols before being transported on ice and analyzed immediately or stored at −80°C until analyzed. Viral genomic DNA was extracted using a TaKaRa Mini BEST Viral RNA/DNA Extraction Kit Ver.5.0 (TaKaRa, Dalian, China), according to the manufacturer’s instructions and then tested for HAdV using the TaqMan real-time PCR kit (Guangzhou HuYanSuo Medical Technology Co., Ltd., Guangzhou, China) as previously reported (Liu et al., 2011). HAdV-positive samples were further molecular typed by PCR amplification of the hypervariable regions of the hexon gene (Lu and Erdman, 2006; Han et al., 2013). This study was authorized by the ethics committee of the First Affiliated Hospital of Guangzhou Medical University, and all participants or their guardians provided written informed consent.

### Virus Isolation

The wild-type strain HAdV-B55-267 was collected in June 2018 from a child who presented with severe ARD and later died in the Guangzhou Women and Children’s Medical Center, China. The wild-type strain HAdV-B55-Shanxi-Y16 isolated in 2011 in Shanxi Province, was kindly provided by Prof. Liqiang Feng (Guangzhou Institutes of Biomedicine and Health, Chinese Academy of Sciences, China), and the wild-type strains HAdV-B55-TY12 and HAdV-B55-TY26 (both isolated in 2013 in Shanxi Province) were provided by Prof. Xiliang Wang (Beijing Institute of Microbiology and Epidemiology, China). The viral strains were cultured in A549 cells at 37°C with 5% (v/v) CO_2_ and maintained under standard conditions in DMEM (Gibco) supplemented with 2% (v/v) fetal bovine serum (FBS) and 100 μg/mL penicillin-streptomycin (Gibco). The cytopathic effect (CPE) of inoculated cells was monitored daily. Cell cultures were harvested when the cells reached almost full CPE.

### Genome Sequencing and Phylogenetic Analysis

The HAdV-B55 type-specific primers (Table 1) used to amplify the hexon, fiber, and penton base genes were designed according to the reference strain HAdV-B55-QZ01 (GenBank NO. KJ883522). The whole genomes of strains HAdV-B55-Shanxi-Y16, HAdV-B55-TY12, HAdV-B55-TY26, and HAdV-B55-267 were sequenced using Sanger sequencing after PCR with primer sets (Supplementary Table 1). The complete genomic sequences were assembled using SeqMan software from the Lasergene package. Multiple sequence alignments and phylogenetic tree construction were performed using Molecular Evolutionary Genetics Analysis (MEGA) version 5.05 (Tamura et al., 2011). The multiple sequence alignments were then revised using Vector NTI version 11.5.2. Phylogenetic trees were constructed by the Neighbor-joining (NJ) method with 1,000 bootstrap replicates, and default settings were used for all other parameters.

**TABLE 1:** Primers for PCR.

**Primer**	**Sequence**
BH49U	GGACAGGATGCTTCGGAGTACCT
BH2813R	GAGAACGGTGTGCGCAGGTAGAC
Ad55hexF	CGGGAGGACAATACATAC
Ad55hexR	GTGGAAAGGCACATAACG
Ad55fibF	CTCCTTCAACCCTGTCTA
Ad55fibR	GTTCCAGGACCAAGTTAT
AdV55penF	GTGGGCAGACAGAATGGA
AdV55penR	CAGGAAAGCGGTTGAAGA

### Micro-Neutralization Test

First, sera were heated at 56°C for 30 min to inactivate the complement. The 50% tissue culture infectious dose (TCID_50_) of the adenoviruses were estimated by the typical method using A549 cells. HAdV-B55 was diluted to 100 TCID_50_/50 μL. The diluted HAdV-B55 solutions were mixed with anti–HAdV-B55 sera and incubated at 37°C for ≥1 h and transferred to 96-well plates containing 70–90% confluent monolayers of A549 cells. The monolayers were cultured for 96 h, after which the infection was evaluated by microscopy, and the neutralization titers were determined as the reciprocal of the highest serum dilution that completely inhibited visually observable CPE.

### Viral Plaque Assays

A549 cells were seeded into 24-well culture plates and incubated overnight to form dense monolayer cells with >90% confluence. After removing the growth medium, the culture was inoculated with 0.2 mL of serial, 10-fold dilutions of the viral stocks and incubated for 1 h at 37°C with rocking every 15 min. The virus inoculums were removed by aspiration, and then 2 mL DMEM-agarose mulch [2% SeaPlaque GTG-agarose (Lonza) mixed 1:1 with 2 × DMEM medium containing 4% FBS] was added to each well. The agarose was allowed to solidify at room temperature (20–26°C). Plaque plates were incubated at 37°C with 5% CO_2_ for 12–14 days and then stained with 1 mL/well of 20% ethanol, 2% paraformaldehyde, and 1% crystal violet overnight at room temperature. Afterward, the plaques were counted, and plaque-forming titers were calculated in PFU/mL.

### Sequences Used in the Study

The HAdV sequences of the hexon and fiber genes and the genomes for phylogenetic analyses retrieved from GenBank are summarized in Table 2. Additional available sequence origin details (strain names, collection date, countries, and GenBank accession numbers) are also included.

**TABLE 2:** The genome, hexon, and fiber sequences of adenovirus species B used for reference in this study.

**Type**	**Strain**	**Collection date**	**Country**	**Sequence**	**GenBank no.**
HAdV-B3	GB	2004	United States	Genome	AY599834
HAdV-B3	HADV-3	2005	Switzerland	Genome	DQ086466
HAdV-B7	HADV-7 vaccine	2004	United States	Genome	AY594256
HAdV-B7	HADV-7	2005	United States	Genome	AC_000018
HAdV-B16	ch. 79	2004	United States	Genome	AY601636
HAdV-B21	VRDL T98-1269	1998	United States	Genome	KJ364592
HAdV-B21	AV-1645	2004	United States	Genome	AY601633
HAdV-B66	87-922	1987	Argentina	Genome	JN860676
HAdV-B68	Arg 827/04	2004	Argentina	Genome	JN860678
HAdV-B34	Compton	2004	United States	Genome	AY737797
HAdV-B35	HADV-35p	2003	Netherlands	Genome	AY271307
HAdV-B35	HADV-35	2004	United Kingdom	Genome	AC_000019
HAdV-B50	Wan	2005	United States	Genome	AY737798
HAdV-B76	DEU/HEIM_00086/X/X[PXHXFX]	2013	Germany	Genome	KF633445
HAdV-B77	DEU/HEIM_00092/1985/NEW[P35H34F7	2013	Germany	Genome	KF268328
HAdV-B78	CHOP2146-10810/2013/[P11H11F7]	2013	United States	Genome	KT970441
HAdV-B79	T150125/2015/79[P11H34F11]	2015	Japan	Genome	LC177352
HAdV-B11	Slobitski	2002	Sweden	Genome	AF532578
HAdV-B11p	Strain 11p	2004	Netherlands	Genome	AY598970
HAdV-B14	de Wit	1955	Netherlands	Genome	AY803294
HAdV-B14	CHN 2012	2012	China	Genome	JX892927
HAdV-B11/14	Spain/273	1969	Spain	Genome	MN654395
HAdV-B11a	EGY/ak37	2001	Egypt	Genome	JX423385
	ARG/ak36	2005	Argentina	Genome	JX423384
	SGN1222	2005	Singapore	Genome	FJ597732
HAdV-B55	CADOH VRDL 76-0669	1976	United States	Genome	MN654394
	CDC97026382	1997	United States	Genome	MN654392
	NAMRU3-E3	2000	Egypt	Genome	MN654380
	NAMRU3-E66	2002	Egypt	Genome	MN654382
	QS-DLL	2006	China	Genome	FJ643676
	WPAFB24	2009	South Korea	Genome	MN654379
	CQ-814	2010	China	Genome	JX123027
	Shanxi-Y16	2011	China	Genome	MK123979
	BJ01	2011	China	Genome	JX491639
	CQ-1657	2011	China	Genome	JX123028
	Shanxi/QZ01	2011	China	Genome	KJ883522
	WPAFB415	2012	Japan	Genome	MN654393
	CQ-2903	2012	China	Genome	JX123029
	Hebei/BD01	2012	China	Genome	KP896478
	AQ-1	2012	China	Genome	KP279748
	XZ2012-492	2012	China	Genome	KC857701
	TY12	2013	China	Genome	MK123980
	TY26	2013	China	Genome	MK123981
	TJ-2013-90	2013	China	Genome	KF908851
	Tianjin/TJ01	2013	China	Genome	KP896484
	Liaoning/LS01	2013	China	Genome	KP896483
	Hebei/BD6728	2013	China	Genome	KJ883520
	Hebei/BD6729	2013	China	Genome	KJ883521
	JS201501	2015	China	Genome	KX289874
	Tibet/LS89	2016	China	Genome	KY002683
	AFMC 16-0011	2016	South Korea	Genome	KX494979
	Sichuan/SF04	2016	China	Genome	KY002684
	Yunnan/KM04	2016	China	Genome	KY002685
	60-GD-2016	2016	China	Genome	KY070248
	267	2018	China	Genome	MK123978
HAdV-B11a	South Dakota/6380	1997	United States	Hexon	FJ841899
	Taiwan/2474	2001	China	Hexon	FJ841906
	Taiwan/760	2002	China	Hexon	FJ841905
	RKI-2797/04	2004	Turkey	Hexon (partial)	AY972815
	SNG 1218	2005	Singapore	Hexon (partial)	FJ607010
	SNG 1222	2005	Singapore	Hexon	FJ841904
HAdV-B55	SHX-P01	2011	China	Hexon	KC999882
	AH-CHN/CZ-TC8	2012	China	Hexon	KC551973
	HAdv-Israel-3927-13	2013	Israel	Hexon (partial)	KR606387
	HAdv-Israel-3957-13	2013	Israel	Hexon (partial)	KR606388
	HAdv-Israel-3958-13	2013	Israel	Hexon (partial)	KR606389
	BJ10	2013	China	Hexon	KM458628
	HAdV-D.IQ1	2013	Iraq	Hexon (partial)	KR914483
	HAdv-Israel-3958-13	2013	Israel	Hexon (partial)	KR606389
	SD77001	2014	China	Hexon	KR912178
HAdV-B11a	Spain/273	1969	Spain	Fiber	FJ841908
	South Dakota/6380	1997	United States	Fiber	FJ841907
	Taiwan/2474	2001	China	Fiber	FJ841914
	Taiwan/760	2002	China	Fiber	FJ841913
	RKI-2797/04	2004	Turkey	Fiber (partial)	AY972816
	SNG 1223	2005	Singapore	Fiber	FJ603104
	SNG 1218	2005	Singapore	Fiber	FJ603105
	SNG 1222	2005	Singapore	Fiber	FJ841912

## Results

### Detection of HAdV-B55 Among Patients With ARD

In this study, 3,191 hospitalized pediatric patients with ARD were enrolled between May 2017 and April 2019. The median age of the patients was 1.58 years (interquartile range, 0.67–4.00) and the male to female ratio was 2.04:1 (2143:1048). Of the 3,191 patients, 159 (4.9%) tested positive for HAdV with 11 of the 159 (6.9%) testing positive for HAdV-55. Only one sample testing positive for HAdV-B55 was isolated during the first year of study period (May 2017–April 2018). The HAdV-B55 positive rate significantly increased after May 2018 (0.56–0.07%) (*P* < 0.05 by χ^2^ test) (Table 3).

**TABLE 3:** HAdV-B55 detected in hospitalized children with ARD in Guangzhou.

**Span of sample collected**	**HAdV positive**	**HAdV-B55 positive**
May 2017 – April 2018 (*n* = 1399)	45 (3.22%)	1 (0.07%)
May 2018 – April 2019 (*n* = 1792)	114 (6.36%)	10 (0.56%)
Total (3191)	159 (4.98%)	11 (0.34%)

### Characterization of HAdV-B55 Strains *in vitro*

**FIGURE 1:**
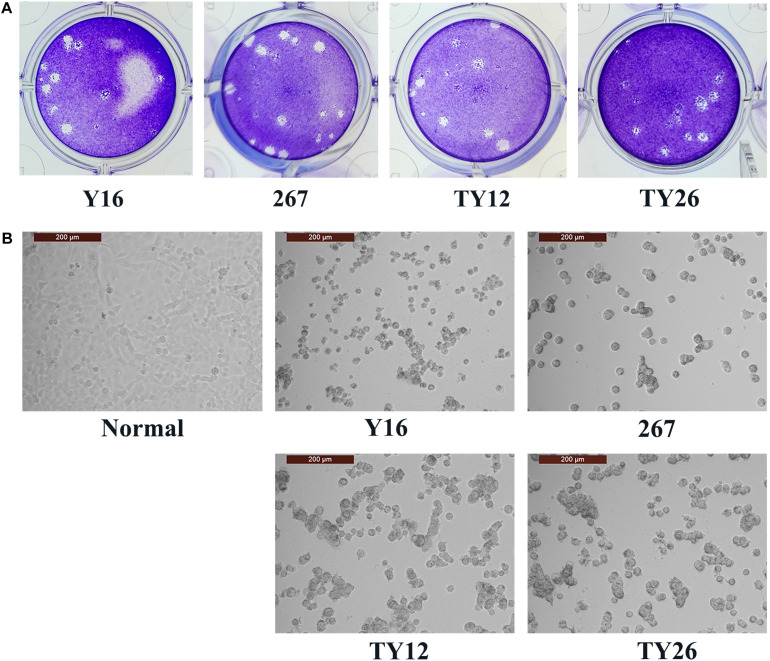
Plaque-forming units in A549 cells infected with HAdV-B55. **(A)** The plaques of HAdV-B55-Shanxi-Y16, HAdV-B55-TY12, HAdV-B55-TY26, and HAdV-B55-267 isolates formed on day 12–14 post-infection of A549 cells. **(B)** The CPE phenotype of A549 cells 48 h after infection with HAdV-B55-Shanxi-Y16, HAdV-B55-TY12, HAdV-B55-TY26, and HAdV-B55-267 isolates at an MOI of 2 PFU/cell.

Four HAdV-B55 strains, HAdV-B55-Shanxi-Y16, HAdV-B55-TY12, HAdV-B55-TY26, and HAdV-B55-267 were successfully isolated from clinical samples and cultured. All four HAdV-B55 strains could be neutralized by mouse anti–HAdV-B55-Shanxi-Y16 sera to homologous or similar high titers in neutralizing tests. The HAdV-B55 strains had similarly high titers (approximately 10^–9^ PFU/mL) and large plaques. The A549 cells showed similar typical CPE 48 h after being infected by the four strains at a multiplicity of infection (MOI) of 2 PFU/cell (Figure 1).

### Phylogenetic Analysis of the Whole Genomes, the Hexon, and Fiber Proteins of the HAdV-B55 Strains

**FIGURE 2:**
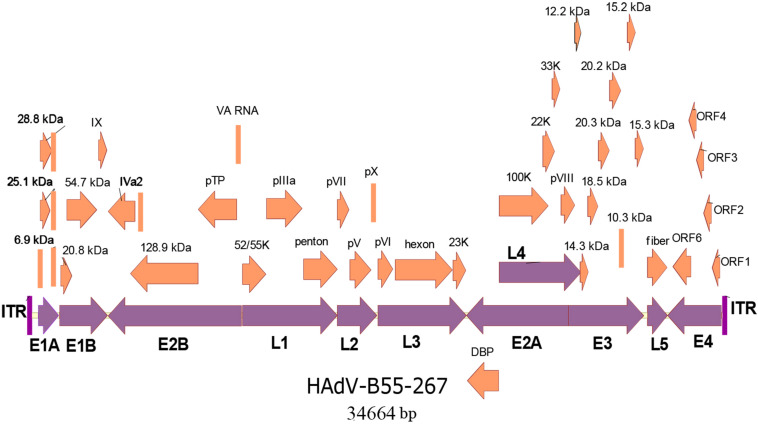
Transcriptional map and genomic organization of strain HAdV-B55-267. Early, intermediate, and late transcription units are designated by purple arrows, whereas orange color designates coding regions. Arrows show the transcriptional orientation of the coding transcripts.

The whole genomes of the four HAdV-B55 strains were sequenced. The genomic data of strain HAdV-B55-267 were deposited into GenBank (GenBank NO. MK123978) with the formal name of “Human adenovirus 55 strain 267,” further referred to as “267.” The genomic data of strains HAdV-B55-Shanxi-Y16, HAdV-B55-TY12, and HAdV-B55-TY26 were also deposited into GenBank with the accession numbers MK123979, MK123980, and MK123981, respectively. Figure 2 presents the genomic organization and transcription map of strain HAdV-B55-267. The genome was 34,664 bp long and contained four early, two intermediate, and five late transcription units. It was composed of 26.13% A, 25.05% T, 24.41% G, and 24.41% C, with a GC content of 48.82%, which was similar to other B2 subspecies (mean of 49%)(Seto et al., 2010).

**FIGURE 3:**
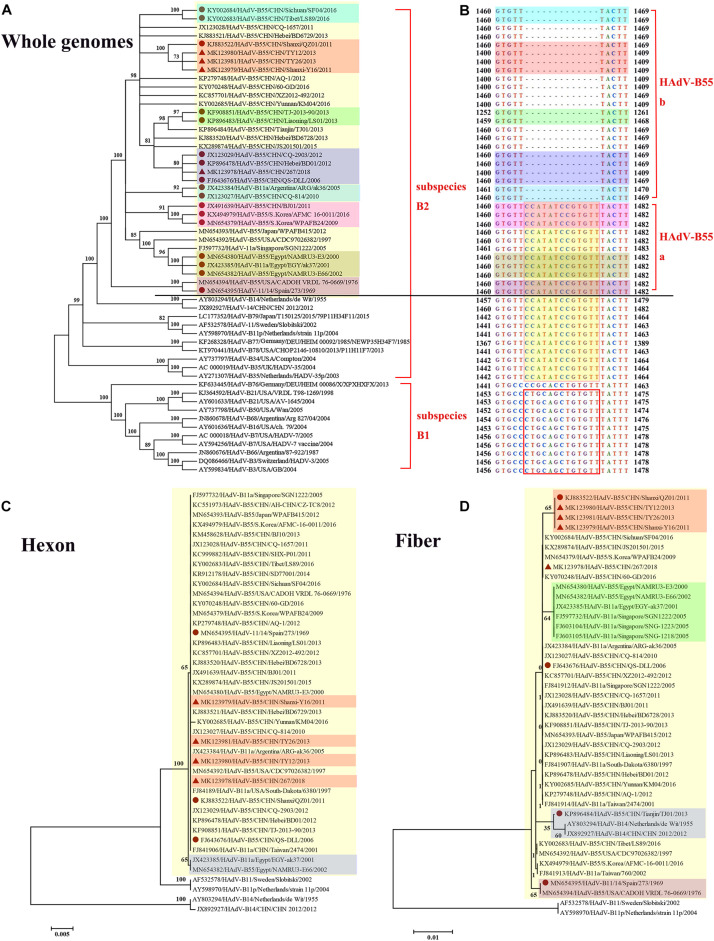
Phylogenetic analysis of HAdV-B55. The phylogenetic trees based on the whole genomic sequence of adenovirus species B **(A)** as well as further genomic analysis aligned with the corresponding sequences **(B)**, and hexon **(C)** or fiber **(D)** amino acid sequence. HAdV-B includes two subspecies, B1 and B2 **(A)**. HAdV-B55 was divided into two subtypes (HAdV-B55a and HAdV-B55b) according to the presence or absence of the insertions of “CCATATCCGTGTT,” located in the non-coding region of the upstream of E1A poly(A) region **(B)**. Bootstrapped, neighbor-joining trees with 1,000 replicates were constructed using the MEGA 5.05 software with default parameters, and a neighbor-joining method. Bootstrap numbers shown at the nodes indicate the percentages of the 1,000 replications producing the clade. The scale bar indicates the units of nucleotide substitutions per site. The strains sequenced or concerned in this study are labeled with red solid triangles and circles, respectively. The sequences used for phylogenic analysis were retrieved from GenBank and are summarized in Table 2.

Whole-genome phylogenetic analysis showed that the four adenoviruses sequenced in this study were of the HAdV-B55 subtype (Figure 3A), corroborating the neutralizing assay results. The phylogenetic tree of the whole genomes revealed that strains HAdV-B55-267, QS-DLL, CQ-2903 (GenBank NO. JX123029), and BD01 (GenBank NO. KP896478) formed a subclade, confirming their close relationship. Furthermore, strains HAdV-B55-Shanxi-Y16, HAdV-B55-TY12, and HAdV-B55-TY26 were closely related to strain QZ01. It is worth noting that strain BJ01 (GenBank NO. JX491639), isolated in Beijing, China, and strain AFMC16-0011 (GenBank NO. KX494979) as well as strain WPAFB24 (GenBank NO. MN654379), both isolated in South Korea, formed a subclade; except for strain BJ01, all HAdV-B55 strains in China are in an evolutionary cluster. In addition, strain CQ-814 (GenBank NO. JX123027), isolated in 2010 in China, and strain ARG-ak36 (GenBank NO. JX423384), isolated in 2005 in Argentina, formed another subclade. Further genomic analysis of HAdV-B55 strains found that, HAdV-B55 strains represented by the earliest strain Spain/273/1969, and the original parental types HAdV-B14 and HAdV-B11, had a nucleotide sequence insertion of “CCATATCCGTGTT,” located in the non-coding region (NCR) of the upstream of E1A poly(A) region (Figure 3B). Interestingly, all types of subspecies B2 (HAdV-B11, -14, -34, -35, -77, -78, and -79) also have this insertion which are highly conserved in this subspecies. All types of subspecies B1 (HAdV-B3, -7, -16, -21, -50, -66, and -68) had a different nucleotide sequence insertion of “CTGCAGCTGTGTT” in this region (Figure 3B). However, except for strain BJ01 isolated in 2011, all HAdV-B55 strains found in China so far including the four adenoviruses sequenced in this study did not have this “CCATATCCGTGTT” insertion. It is speculated that the HAdV-B55 strains in China except strain BJ01 have a common source with strain ARG-ak36 isolated in Argentina in 2005, while strain BJ01 may originate from outside of China, such as Korea. The HAdV-B55 strains from Asia-Pacific region including Japan, South Korea and Singapore may have a common source.

The HAdV capsid is composed of three major proteins, the hexon, fiber, and penton base. The hexon protein is the predominant target of neutralizing antibodies, and the fiber protein mediates the specific, high-affinity binding to the primary cellular receptors. The phylogenetic tree based on the hexon amino acid sequences showed that all the HAdV-B55 strains, including strain Spain/273/1969 isolated in 1969 (GenBank NO. MN654395), formed a subclade, except for strains EGY-ak37 (GenBank NO. JX423385) and NAMRU3-E66 (GenBank NO. MN654382), both isolated in Egypt (Figure 3C). The hexon proteins of the HAdV-B55 strains recently isolated in China and strain Spain/273/1969 noticeably clustered in the same branch. In the fiber region, strains HAdV-B55-Shanxi-Y16, HAdV-B55-TY12, and HAdV-B55-TY26 formed a subclade with strain QZ01, corresponding to the phylogenetic tree of whole genomes (Figure 3D). Amino acid sequence alignment previously indicated that HAdV-B55 shares the fiber knob with HAdV-B14p but not with HAdV-B14p1 (Tian et al., 2018). Furthermore, those strains isolated in Egypt and Singapore formed a subclade. Interestingly, the fiber protein of strain TJ01 (GenBank NO. KP896484) formed a subclade with the subclade of HAdV-B14p1 strains. Additionally, the phylogenetic tree based on the penton base amino acid sequences indicated no particular difference among the strains (data not shown).

### Comparative Genomic Analysis of HAdV-B55 Strains

To assess the global genomic mutation map of the alignments, each point mutation, as well as synonymous (dS) and non-synonymous (dN) substitutions (Nei and Gojobori, 1986) were identified and compared using strain QS-DLL as the reference (Table 4). Compared with strain QS-DLL, strain CQ-2903 had 7 mutations, including 4 synonymous substitutions and 3 indels in the NCR; strain HAdV-B55-267 had 10 mutations, including 5 synonymous substitutions, 1 non-synonymous substitution located in the coding region of L1 pIIIa 65.6 kDa Protein, 3 indels located in the NCR, and a G-to-A mutation in E1B NCR; strain BD01 had 10 mutations, including 3 synonymous, 2 non-synonymous substitutions located in the genes of L1 pIIIa 65.6 kDa Protein and L2 pV 40.1 kDa Protein, 4 indels and a G-to-A mutation in E1B NCR; and strain 60-GD had 13 mutations, including 6 synonymous, 2 non-synonymous substitutions located in the genes of 20.8 kDa/20 kDa Protein and 54.7 kDa/54.9 kDa Protein, 4 indels and an A-to-G mutation in E4 NCR. Interestingly, strain Spain/273/1969 had 61 mutations, including 28 synonymous substitutions, 17 non-synonymous substitutions, 9 indels located in the NCR, 6 mutations in the NCR, and a G-to-C mutation in the inverted terminal repeat (ITR). The 17 non-synonymous substitutions were located in the coding region of E1B 20.8 kDa Protein, E1B 54.7 kDa Protein, E2B 128.9 DNA polymerase, pTP, L4 21.6 kDa Protein, E3 20.3 kDa Protein, E3 10.3 kDa Protein, E3 15.3 kDa Protein, L5 fiber 35.3 kDa Protein, E4 Orf6/7, E4 ORF6 34.6 kDa Protein, and E4 ORF4 14.2 kDa Protein. Furthermore, the capsid protein genes were highly conserved between strain QS-DLL and the four HAdV-B55 strains. Both the hexon and fiber genes of these five HAdV-B55 strains were identical to each other. There was only one single-nucleotide non-synonymous substitution (D-to-H) in the fiber gene of strain Spain/273/1969.

**TABLE 4:** Comparative genomic analysis of HAdV-B55 strains 267, 60-GD, BD01, CQ-2903, Y16, TY12, TY26, and QZ01 with the reference strain QS-DLL.

**Region**	**Gene**	**Mutation in DNA**										**Mutation in AA (dN)**							
		**273**	**267**	**60-GD**	**BD01**	**CQ-2903**	**Y16**	**QZ01**	**TY12**	**TY26**	**273**	**267**	**60-GD**	**BD01**	**CQ-2903**	**Y16**	**QZ01**	**TY12**	**TY26**
E1A	NCR	T → G	–	–	–	–	–	–	–	–	–	–	–	–	–	–	–	–	–
		C → T	–	–	–	–	–	–	–	–	–	–	–	–	–	–	–	–	–
		A → G	–	–	–	–	–	–	–	–	–	–	–	–	–	–	–	–	–
		C → G	–	–	–	–	–	–	–	–	–	–	–	–	–	–	–	–	–
	28.8 kDa	–	–	–	C → T	–	–	–	–	–	–	–	–	dS	–	–	–	–	–
		–	–	C → T	–	–	–	–	–	–	–	–	dS	–	–	–	–	–	–
		C → T	–	–	–	–	–	–	–	–	dS	–	–	–	–	–	–	–	–
	NCR	▼CCATATCCGTGTT	–	–	–	–	–	–	–	–	–	–	–	–	–	–	–	–	–
E1B	NCR	–	G → A	–	–	–	–	–	–	–	–	–	–	–	–	–	–	–	–
	20.8 kDa/20 kDa	A → G	–	–	–	–	–	–	–	–	N → S	–							
		A → T	–	A → T	–	–	A → T	A → T	A → T	A → T	I → L	–	I → L	–	–	I → L	I → L	I → L	I → L
		T → C	–	–	–	–	–	–	–	–	dS	–	–	–	−	−	−	−	−
	54.7 kDa/54.9 kDa	T → C	−	T → C	−	−	T → C	T → C	T → C	T → C	S → P	−	S → P	−	−	S → P	S → P	S → P	S → P
		C → T	−	−	−	−	−	−	−	−	S → F	−	−	−	−	−	−	−	−
		A → G	−	−	−	−	−	−	−	−	dS	−	−	−	−	−	−	−	−
		A → G	−	−	−	−	−	−	−	−	dS	−	−	−	−	−	−	−	−
	NCR	–	–	–	G → A	–	–	–	–	–	–	–	–	–	–	–	–	–	–
	pIX 14.2 kDa	T → C	–	–	–	–	–	–	–	–	dS	–	–	–	–	–	–	–	–
		–	–	–	–	–	C → T	C → T	C → T	C → T	–	–	–	–	–	S → F	S → F	S → F	S → F
E1B	NCR	▼AAAAAAAAA	–	–	–	–	–	–	–	–	–	–	–	–	–	–	–	–	–
E2B	pIVa2 50.9 kDa	C → T	–	–	–	–	–	–	–	–	dS	–	–	–	–	–	–	–	–
		A → G	–	–	–	–	–	–	–	–	dS	–	–	–	–	–	–	–	–
	DNA polymerase	G → A	–	–	–	–	–	–	–	–	dS	–	–	–	–	–	–	–	–
		–	C → G	–	–	–	–	–	–	–	–	dS	–	–	–	–	–	–	–
		–	–	–	–	G → A	–	–	–	–	–	–	–	–	dS	–	–	–	—-
		C → T	–	–	–	–	–	–	–	–	E → K	–	–	–	–	–	–	–	–
		C → T	–	–	–	–	–	–	–	–	dS	–	–	–	–	–	–	–	–
		T → C	–	–	–	–	–	–	–	–	E → G	–	–	–	–	–	–	–	–
		C → T	–	–	–	–	–	–	–	–	dS	–	–	–	–	–	–	–	–
	pTP	C → T	–	–	–	–	–	–	–	–	A → T	–	–	–	–	–	–	–	–
		A → G	–	–	–	–	–	–	–	–	dS	–	–	–	–	–	–	–	–
		G → A	–	–	–	–	–	–	–	–	dS	–	–	–	–	–	–	–	–
	NCR	–	–	–	–	–	▲T	–	▲T	▲T	–	–	–	–	–	–	–	–	–
	NCR	G → T	–	–	▼T	–	–	–	–	–	–	–	–	–	–	–	–	–	–
	NCR	▼TTTTT	–	–	–	–	–	–	–	–	–	–	–	–	–	–	–	–	–
L1	43.9 kDa	–	–	A → G	–	–	–	–	–	–	–	–	dS	–	–	–	–	–	–
		G → A	–	–	–	–	–	–	–	–	dS	–	–	–	–	–	–	–	–
	pIIIa 65.6 kDa	–	G → A	–	–	–	–	–	–	–	–	M → I	–	–	–	–	–	–	–
		–	–	–	G → C	–	–	–	–	–	–	–	–	D → H	–	–	–	–	–
	polyA signal	–	–	▼A	–	–	–	–	–	–	–	–	–	–	–	–	–	–	–
L2	Penton 62.5 kDa	–	–	G → A	–	–	–	–	–	–	–	–	dS	–	–	–	–	–	–
		–	G → A	–	–	–	–	–	–	–	–	dS	–	–	–	–	–	–	–
	pV 40.1 kDa	–	–	–	C → T	–	–	–	–	–	–	–	–	T → I	–	–	–	–	–
	NCR	▲A	▼A	▲A	▲A	–	–	–	–	–	–	–	–	–	–	–	–	–	–
	NCR	▼A	–	▼A	▼A	▼A	▼A	–	▼A	▼A	–	–	–	–	–	–	–	–	–
L3	Hexon	G → T	–	–	–	–	–	–	–	–	dS	–	–	–	–	–	–	–	–
		C → T	–	–	–	–	–	–	–	–	dS	–	–	–	–	–	–	–	–
		C → A	–	–	–	–	–	–	–	–	dS	–	–	–	–	–	–	–	–
	23.7 kDa	C → T	–	–	–	–	–	–	–	–	dS	–	–	–	–	–	–	–	–
E2A	DNA-binding protein	–	C → T	–	–	–	–	–	–	–	–	dS	–	–	–	–	–	–	–
		–	–	–	–	A → G	–	–	–	–	–	–	–	–	dS	–	–	–	–
		A → C	–	A → C	–	–	A → C	A → C	A → C	A → C	dS	–	dS	–	–	dS	dS	dS	dS
		A → T	–	–	–	–	–	–	—-	–	dS	–	–	–	–	–	–	–	–
L4	100K 91 kDa	–	–	–	–	–	G → A	G → A	G → A	G → A	–	–	–	–	–	S → N	S → N	S → N	S → N
		A → G	–	A → G	–	–	A → G	A → G	A → G	A → G	dS	–	dS	–	–	dS	dS	dS	dS
	21.6 kDa	G → A	–	–	–	–	–	–	–	–	G → D	–	–	–	–	–	–	–	–
		C → G	–	–	–	–	–	–	–	–	S → W	–	–	–	–	–	–	–	–
	pVIII 25 kDa	C → A	–	–	–	–	–	–	–	–	dS	–	–	–	–	–	–	–	–
		A → C	–	–	–	–	–	–	–	–	dS	–	–	–	–	–	–	–	–
		–	–	–	–	C → T	–	–	–	–	–	–	–	–	dS	–	–	–	–
E3	12.2 kDa/11.7 kDa	T → C	–	–	–	–	–	–	–	–	dS	–	–	–	–	–	–	–	–
		–	–	–	–	–	T → A	–	T → A	T → A	–	–	–	–	–	H → Q	–	H → Q	H → Q
	18.5 kDa	C → T	–	–	–	–	–	–	–	–	dS	–	–	–	–	–	–	–	–
	20.3 kDa/20.1 kDa	C → A	–	–	–	–	–	–	–	–	P → T	–	–	–	–	–	–	–	–
		G → A	G → A	G → A	G → A	G → A	G → A	G → A	G → A	G → A	dS	dS	dS	dS	dS	dS	dS	dS	dS
	NCR	▲TT	▲TT	–	▲T	▲TT	–	–	▼T	▼T	–	–	–	–	–	–	–	–	–
	10.3 kDa	A → T	–	–	–	–	–	–	–	–	Q → H	–	–	–	–	–	–	–	–
	15.2 kDa/14.9 kDa	C → A	–	–	–	–	–	–	–	–	dS	–	–	–	–	–	–	–	–
		–	–	–	G → A	–	–	–	–	–	–	–	–	dS	–	–	–	–	–
	15.3 kDa	C → G	–	–	–	–	–	–	–	–	dS	–	–	–	–	–	–	–	–
		A → G	–	–	–	–	–	–	–	–	N → D	–	–	–	–	–	–	–	–
	NCR	▲A	–	–	–	–	–	–	–	–	–	–	–	–	–	–	–	–	–
	polyA signal	▼A	–	–	–	–	–	–	–	–	–	–	–	–	–	–	–	–	–
L5	Fiber 35.3 kDa	G → C	–	–	–	–	–	–	–	–	D → H	–	–	–	–	–	–	–	–
		–	–	–	–	–	A → C	A → C	A → C	A → C	–	–	–	–	–	T → P	T → P	T → P	T → P
E4	Orf6/7	–	–	–	–	–	G → T	G → T	G → T	G → T	–	–	–	–	–	dS	dS	dS	dS
		G → A	–	–	–	–	–	–	–	–	P → S	–	–	–	–	–	–	–	–
	ORF6 34.6 kDa	–	A → C	–	–	–	–	–	–	–	–	dS	–	–	–	–	–	–	–
		T → C	–	–	–	–	–	–	–	–	N → D	–	–	–	–	–	–	–	–
		T → C	–	–	–	–	–	–	–	–	K → R	–	–	–	–	–	–	–	–
	ORF4 14.2 kDa	C → T	–	–	–	–	–	–	–	–	G → D	–	–	–	–	–	–	–	–
	ORF3 13.6 kDa	A → G	–	–	–	–	–	–	–	–	dS	–	–	–	–	–	–	–	–
	NCR	▲A	▲A	▲A	–	▲A	–	–	–	–	–	–	–	–	–	–	–	–	–
	NCR	C → T	–	–	–	–	–	–	–	–	–	–	–	–	–	–	–	–	–
	NCR	–	–	A → G	–	–	–	–	–	–	–	–	–	–	–	–	–	–	–
	NCR	–	–	–	–	–	–	–	–	G → A	–	–	–	–	–	–	–	–	–
	ITR	G → C	–	–	–	–	–	–	–	–	–	–	–	–	–	–	–	–	–

*Differences in nucleic acid and amino acid sequences are shown along with their genomic locations and coding consequences. The reference genome is HAdV-B55 strain QS-DLL. NCR, non-coding region; ▼, insertion; ▲, deletion; –, no change or not applicable; dS, synonymous; dN, non-synonymous.*

When compared with strain QS-DLL, strains HAdV-B55-Shanxi-Y16, HAdV-B55-TY12, and HAdV-B55-TY26 were highly homologous to strain QZ01, in line with the phylogenetic analysis, except for some small differences. For example, relative to the genome of strain QZ01, one non-synonymous substitution (H-to-Q) was identified in the genes of E3 12.2 kDa/11.7 kDa Proteins of strains HAdV-B55-Shanxi-Y16, HAdV-B55-TY12, and HAdV-B55-TY26, in addition to one G-to-A mutation in E4 NCR of strain HAdV-B55-TY26. Furthermore, compared with the genome of strain QZ01, strain HAdV-B55-Shanxi-Y16 had a nucleotide deletion (T) and insertion (A), strains HAdV-B55-TY12 and HAdV-B55-TY26 had two insertions (A and T, respectively) and one deletion (T). However, both the insertions and deletions were located in the NCR. The capsid protein genes were highly conserved among the four strains. There was one single-nucleotide mutation (A-to-C) in the fiber gene, resulting in a non-synonymous substitution (T-to-P). The three strains were identical in both the penton base and hexon gene sequences.

## Discussion

**FIGURE 4:**
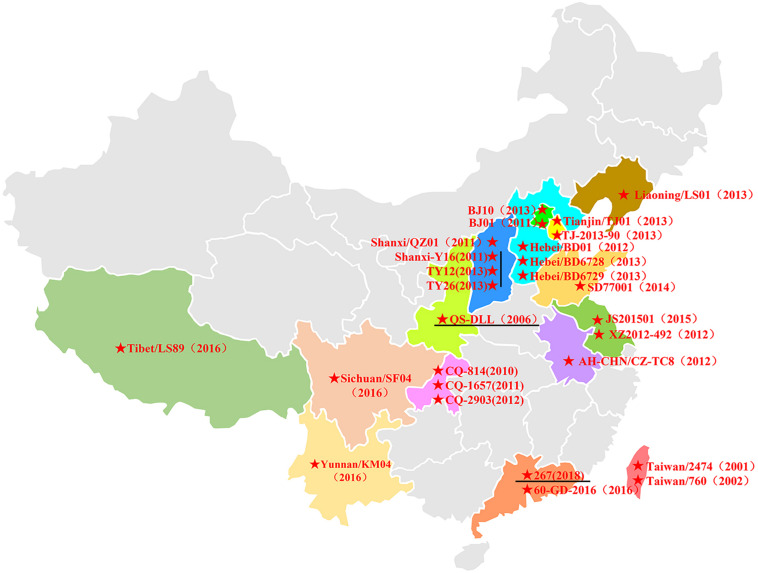
The distribution of HAdV-B55 in China. The HAdV-B55 strains are labeled with solid red stars. The sequences were retrieved from GenBank and are summarized in Table 2.

In China, HAdV-B55 strain QS-DLL was first identified in 2006 from an outbreak in a senior high school in Qishan County of Shaanxi Province (Yang et al., 2009; Zhu et al., 2009; Walsh et al., 2010). Since 2010, sporadic or outbreak cases of HAdV-B55 infections have increasingly been reported, for example, in Chongqing, Shanxi, Hebei, Liaoning, Beijing, Anhui, and Jiangsu. In 2016, several HAdV-B55 outbreaks were reported in Xizang, Yunnan, and Sichuan (Wang et al., 2017) and Guangdong (Yi et al., 2017), suggesting that this recombinant adenovirus was widely disseminated in China (Figure 4). Since then, few cases of HAdV-B55 were reported. Here, we found only one patient was infected by HAdV-B55 during the 12 months prior to May 2018; thereafter, ten HAdV-B55-positive patients were detected in one year in Guangzhou, a central city in southern China. This indicates HAdV-B55 is an important pathogen in the general child population in Guangzhou and more attention should be paid to this.

Here, HAdV-B55 whole genomes available in NCBI are presented in the highest possible detail, and a whole-genome phylogenetic tree was constructed to elucidate the evolution and variation of HAdV-B55 in China over the past decades. Accordingly, the closest genetic relationship was found among the 2018 Guangdong strain (HAdV-B55-267), 2012 Chongqing strain (CQ-2903), 2012 Hebei strain (BD01), and 2006 Shaanxi strain (QS-DLL). Further comparative genomic analyses were performed to verify this observation (Table 4). Lu et al. (2014) have also shown in detail that strain CQ-2903 is closely related to QS-DLL. Furthermore, strain 60-GD also appeared in Guangdong Province and had only two non-synonymous substitutions (Table 4). These viruses merely had small mutations, indicating that the pathogens might be of the same origin. Moreover, this study showed that strains HAdV-B55-Shanxi-Y16, HAdV-B55-TY12, and HAdV-B55-TY26 were closely related to strain QZ01, having similar mutation patterns and indicating that the pathogens causing these outbreaks might be of the same origin. Furthermore, the phylogenetic tree based on the whole genomes showed that strains BJ01 isolated in 2011 in Beijing, AFMC16-0011 and WPAFB24 isolated in Korea in 2016 and 2009, respectively, formed a subclade that included 16 single-nucleotide substitutions. The strains CQ-814 isolated in 2010 in China and ARG-ak36 isolated in 2005 in Argentina formed another subclade, which included 17 single-nucleotide substitutions (data not shown).

We proposed that HAdV-B55 could be divided into two subtypes (HAdV-B55a and HAdV-B55b) according to the presence or absence of the insertions of “CCATATCCGTGTT” (Figure 3B). This is coincident with the whole-genome phylogenetic analysis of HAdV-B55 strains (Figure 3A). The phylogenetic tree of E1A gene including genomic nucleotide 435–1,450 bp (HAdV-B55 strain 267 as the control), was also constructed with the option of “partial deletion” for the gaps treatment, which is totally coincident with the whole genome tree with little divergences (Supplementary Figure 1). We strikingly found that, almost all HAdV-B55 strains from outside of China represented by strain Spain/273/1969 had a nucleotide sequence insertion of “CCATATCCGTGTT,” located in the NCR of the upstream of E1A poly (A) region. However, all HAdV-B55 strains found in China so far, except for strain BJ01, did not have this insertion. Cheng et al. (2018) firstly reported the genomic sequence of HAdV-B55 BJ01 strain associated with adult severe community-acquired pneumonia in Beijing in 2011. Comparative genomic analysis of this re-emergent HAdV-B55 strain (BJ01) with the first HAdV-B55 strain (QS-DLL; 2006) showed the high genome identity (99.87%) (Cheng et al., 2018). Here the comparative genomic analysis of HAdV-B55 strain 267 (2018) with QS-DLL showed the higher genome identity (99.97%). The only strain outside of China which had no this insertion, ARG-ak36, was isolated in Argentina in 2005, a year before the first strain QS-DLL isolated in China. It is speculated that the epidemic HAdV-B55 strains in most regions of China originated from a common source with strain ARG-ak36. Only strain BJ01/2011 may have a different source, which may originate from strain WPAFB24/2009 isolated in South Korea. HAdV-B55b strains may evolve from a strain of HAdV-B55a by illegitimate recombination, resulting in the deletion of this insertion sequence. The illegitimate recombination (i.e., insertion or deletion) is an important and relatively common mechanism of adenovirus rapid evolution which usually happened when HAdV DNA polymerase slides on the genome chain to duplicate purine polymer (Crawford-Miksza and Schnurr, 1996).

Although Li et al. (2014) had reported that strain TJ-2013-90 shared almost 100% homology (with only four different nucleotides) with strain QS-DLL, we found that at least 17 nucleotides were different and the whole genomic sequence was incomplete, missing the sequence of ORF1 14.2 kDa Protein in the E4 region (data not shown). Since the fiber protein of strain TJ01 was closely related to HAdV-B14, we aligned the predicted fiber amino acid sequences of the selected HAdV-B55 strains. Compared to the other HAdV-B55 strains, strain TJ01 had two amino acid substitutions (K116E and I127N). Interestingly, the fiber protein of strain TJ01 lacked two amino acids (250EK251) similar to that of HAdV-B14p1 strains (data not shown). The most notable genetic difference between the variant HAdV-B14p1 and the prototype HAdV-B14p was a 6-bp–long deletion in the fiber knob gene (Kajon et al., 2010b; Tian et al., 2018). Wang et al. (2017) showed that LS89/Tibet/2016 and SF04/SC/2016 are of the same origin, form a subclade (Figure 3), and have at least 16 different nucleotides compared with strain QS-DLL.

Importantly, when compared with strain 267, strains HAdV-B55-Shanxi-Y16, HAdV-B55-TY12, and HAdV-B55-TY26 had one non-synonymous substitution (S-to-N) in L4 100K, which may affect viral growth phenotype, antigenicity, infectivity, or virulence. Nevertheless, our study found that plaque size and morphology and viral titers *in vitro* showed no significant difference among strain HAdV-B55-267 and the three HAdV-B55 strains isolated earlier in northern China, suggesting that these strains do not significantly differ in growth phenotype, infectivity, or virulence. Micro-neutralization test results also indicated no significant divergence in antigenicity of these viruses. Cheng et al. (2018) also reported that no significant difference of replication efficiency among HAdV-B55, -B11, and -B14 was identified in A549, Hela, HEp-2, Hep-G2, and Vero cells. The prevalence and outbreak of HAdV-B55 might be not associated with the virus replication efficiency (Cheng et al., 2018). The HAdV-B55 genomic changes appear to be random and not responsible for more infectious or virulent strains, which may cause more serious risks to human health. However, this study lacks detailed clinical data and evidence of the transmission pathway, which may be essential for understanding disease prevention and diagnosis. Also, given the recombination rates due to mixed infection, the employed Sangers sequencing may detect only the dominant viral variant within a mixed viral population in a given sample, so the need for detecting low-frequency or minority variants should be taken seriously in future study.

In summary, we found HAdV-B55 has been an important common pathogen in Guangzhou since 2018, suggesting its continuous transmission in most regions of China. The currently circulating HAdV-B55 strains in both northern and southern China contain relatively stable genomes that are remarkably similar at the nucleotide level across time and space, having accumulated relatively small genomic changes through indels and base substitutions. These strains also showed similar *in vitro* growth characteristics and virulence. The whole-genome analysis results support that local epidemic viral strains have undergone several low-frequency random mutations in the past decades, but the data are limited. More vigorous population-based surveillance for HAdV-B55 strains should be conducted in the future. A further molecular investigation based on HAdV-B55 of a broader origin might facilitate the understanding of HAdV-B55 dissemination and transmission in China.

## Data Availability Statement

The datasets generated for this study can be found in online repositories. The names of the repository/repositories and accession number(s) can be found below:

https://www.ncbi.nlm.nih.gov/genbank/, MK123978; https://www.ncbi.nlm.nih.gov/genbank/, MK123979; https://www.ncbi.nlm.nih.gov/genbank/, MK123980; and https://www.ncbi.nlm.nih.gov/genbank/, MK123981.

## Ethics Statement

Written informed consent was obtained from the individual(s), and minor(s)’ legal guardian/next of kin, for the publication of any potentially identifiable images or data included in this article.

## Author Contributions

XT planned and designed the study. XT and S-yC drafted the manuscript. S-yC and YC conducted the sequence analysis. S-yC, YX, WL, SQ, and XT performed the experiments. RZ guided the study. All authors read and approved the final manuscript.

## Conflict of Interest

The authors declare that the research was conducted in the absence of any commercial or financial relationships that could be construed as a potential conflict of interest.

## Supplementary Material

Supplementary Figure 1 The phylogenetic tree based on the E1A gene sequence of HAdV-B55 strains. HAdV-B55 strains were classified as HAdV-B55a and HAdV-B55b according to the E1 NCR region with or without the insertion nucleotide fragment “CCATATCCGTGTT.”
