# Supplementary figures and images for: Epidemiology and Genetic Variabilities of Human Adenovirus Type 55 Reveal Relative Genome Stability Across Time and Geographic Space in China
Source: Front Microbiol. 2020 Dec 2;11:606195. doi: 10.3389/fmicb.2020.606195 (PMC7738467; doi:10.3389/fmicb.2020.606195)

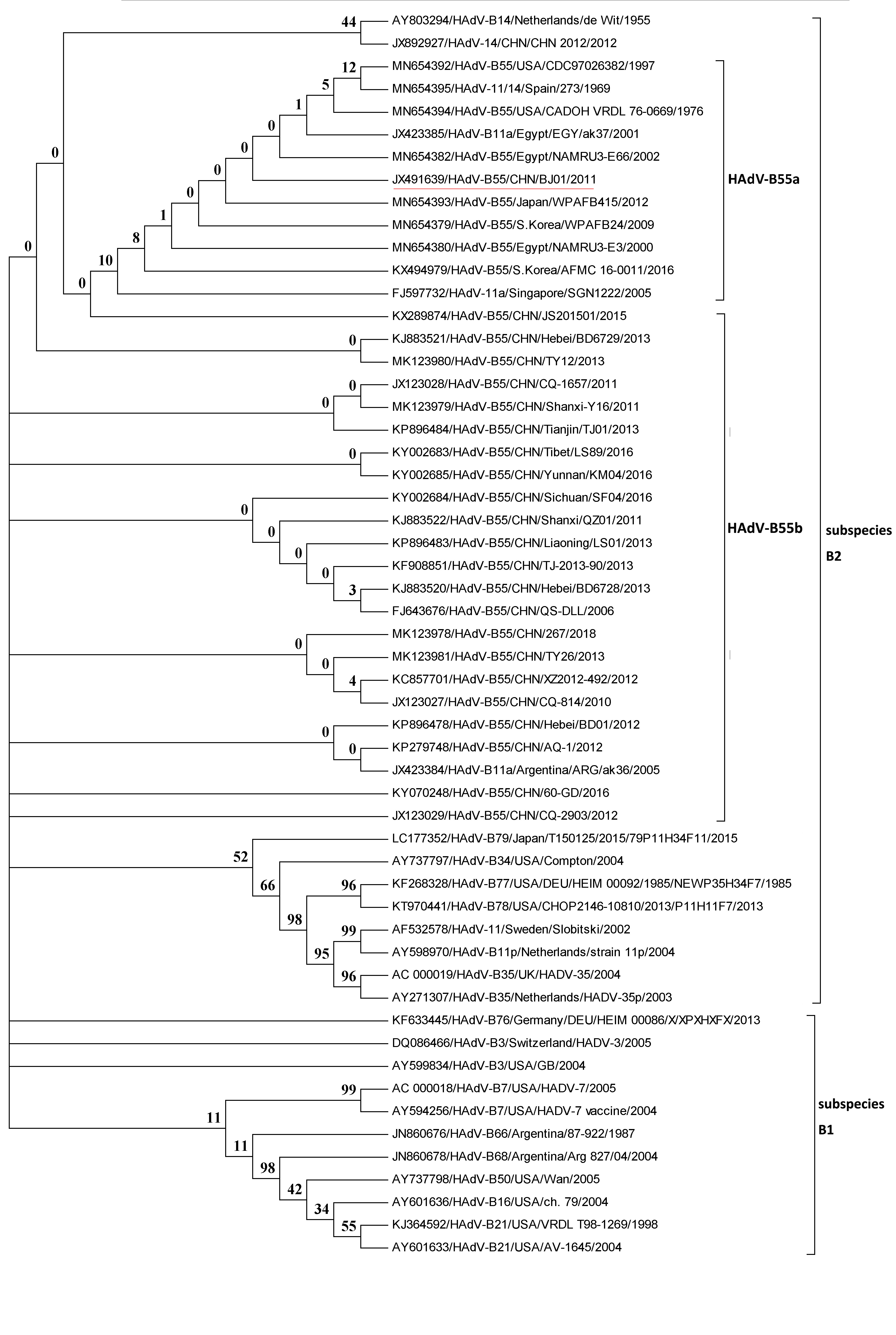

Supplement: Supplementary Figure 1 — The phylogenetic tree based on the E1A gene sequence of HAdV-B55 strains. HAdV-B55 strains were classified as HAdV-B55a and HAdV-B55b according to the E1 NCR region with or without the insertion nucleotide fragment “CCATATCCGTGTT.” [file Image_1.JPEG]
