# Supplementary material for: Epidemiology and Genetic Variabilities of Human Adenovirus Type 55 Reveal Relative Genome Stability Across Time and Geographic Space in China
Source: Front Microbiol. 2020 Dec 2;11:606195. doi: 10.3389/fmicb.2020.606195 (PMC7738467; doi:10.3389/fmicb.2020.606195)
Supplement: Supplementary file 2 [file Table_1.DOCX]

Table S1. Primer sets for HAdV-B55 genome sequencing.

| Primers | Sequences | Amplification position (bases) | Product length |
| --- | --- | --- | --- |
| 10U | TAATATACCTTATAGATGGAATGG | 10-3720 | 3711bp |
| 3720R | CCCATTCCAAGCACAGTGTT |  |  |
| IVaU | TGGGCAGGAGTTCGTCAGAATGT | 3554-6047 | 2494bp |
| IVaR | GTCAATATCAAATCCTCCTCGTT |  |  |
| 5885U | CTGGGGGGGTATAAAAGGG | 5885-11775 | 5891bp |
| 11775R | TCATCCGCCTCTTCTATGTAAGG |  |  |
| 5852U | TGTAGGTGTATTTCACGTGACC | 5852-12016 | 6165bp |
| 12016R | GCTTCCATGATGGCCGATAGACG |  |  |
| 11877U | CTGCAGAGCCAGCCGTCCGG | 11877-15222 | 3346bp |
| 15222R | GTCCCGTGATCTGTGAGAGCAGG |  |  |
| 14995U | CTCCACTAGACAAGTCAGCAACTA | 14995-18470 | 3476bp |
| 18470R | AAGAGTATGTATTGTCCTCCCGGTC |  |  |
| 15200U | CCTGCTCTCACAGATCACGGGAC | 15200-18470 | 3271bp |
| 18470R | AAGAGTATGTATTGTCCTCCCGGTC |  |  |
| BH49U | GGACAGGATGCTTCGGAGTACCT | 18281-21051 | 2771bp |
| BH2813R | GAGAACGGTGTGCGCAGGTAGAC |  |  |
| 20836U | GTTCTCGAGCAACTTCATGTC | 20836-26714 | 5879bp |
| 26714R | TACACTAGGGCGGCAGGCCAATT |  |  |
| BFU | AAGTACTGCGCGCTGACTCTTAA | 26397-32431 | 6035bp |
| BFR | ATGCCAAAAGAGATTATGTATGTGGG |  |  |
| BFU2 | CGAGACGCCCAGGCCGAAGT | 26752-32431 | 5680bp |
| BFR | ATGCCAAAAGAGATTATGTATGTGGG |  |  |
| 32222U | GCAGCAACGCATTCTTATTTCACT | 32222-34746 | 2525bp |
| 34746R | TAATATACCTTATAGATGGAATGGTGC |  |  |
